# The prognostic value of advanced lung cancer inflammation index (ALI) in elderly patients with heart failure

**DOI:** 10.3389/fcvm.2022.934551

**Published:** 2022-11-11

**Authors:** Xiao Yuan, Bi Huang, Ruiyu Wang, Hongtao Tie, Suxin Luo

**Affiliations:** ^1^Department of Cardiology, The First Affiliated Hospital of Chongqing Medical University, Chongqing, China; ^2^Institute of Life Science, Chongqing Medical University, Chongqing, China; ^3^Department of Cardiothoracic Surgery, The First Affiliated Hospital of Chongqing Medical University, Chongqing, China

**Keywords:** heart failure, elderly, nutrition, inflammation, advanced lung cancer inflammation index, mortality

## Abstract

**Purpose:**

The advanced lung cancer inflammation index (ALI) is a novel inflammatory and nutritional index that exerts prognostic value in various types of cancer. A previous study demonstrated that ALI at discharge could predict the prognosis in patients with acute decompensated heart failure (ADHF). However, the long-term prognostic value of ALI on admission in elderly heart failure (HF) inpatients remains unclear.

**Materials and methods:**

We retrospectively collected HF inpatients over 65-year-old who were hospitalized in our cardiology center during the whole year of 2015. ALI was calculated as body mass index (BMI) × serum albumin (Alb)/neutrophil-to-lymphocyte ratio (NLR). Patients were divided into two groups by the optimal cutoff value of ALI for predicting all-cause mortality using time-dependent receiver operating characteristic (ROC) curves. The Spearman rank correlation coefficient was computed to evaluate the correlation between ALI and the geriatric nutritional risk index (GNRI). Kaplan–Meier curves, Cox survival analyses, time-dependent ROC analyses, and net reclassification improvement (NRI) analyses were used to assess the prognostic effect of ALI on all-cause mortality and cardiovascular mortality.

**Results:**

Over a 28-month median follow-up, all-cause and cardiovascular mortality occurred in 192 (35.4%) and 132 (24.3%) out of 543 patients, respectively. The optimal cutoff value of ALI for predicting all-cause mortality at 2 years was 25.8. Spearman’s correlation coefficient showed a moderate positive linear correlation between ALI and GNRI (*r* = 0.44, *p* < 0.001). The Kaplan–Meier analysis revealed that the cumulative incidences of both all-cause and cardiovascular mortalities were significantly higher in patients with lower ALI (log-rank test, all-cause mortality: *p* < 0.0001; cardiovascular mortality: *p* < 0.0001). The multivariate Cox proportional hazard analyses indicated that ALI was an independent predictor for both all-cause mortality (HR 0.550, 95% CI 0.349–0.867, *p* = 0.01) and cardiovascular mortality (HR 0.536, 95% CI 0.302–0.953, *p* = 0.034). Time-dependent ROC analyses showed that ALI was comparable to GNRI in predicting long-term all-cause mortality (AUC: ALI 0.625, GNRI 0.641, *p* = 0.976) and cardiovascular mortality (AUC: ALI 0.632, GNRI 0.626, *p* = 0.999) at 2 years. However, the estimated NRI indicated that the addition of ALI could not significantly improve risk stratification of base models for all-cause mortality (categorical NRI 4.9%, *p* = 0.433, continuous NRI 25%, *p* = 0.022) or cardiovascular mortality (categorical NRI 6.5%, *p* = 0.223, continuous NRI 27.5%, *p* = 0.029).

**Conclusion:**

Higher ALI was significantly associated with lower all-cause and cardiovascular mortalities in elderly HF patients. ALI on admission could be a competent nutrition-inflammation marker with independent predictive value for evaluating the long-term mortality of HF in elder population.

## Introduction

Heart failure (HF) has been regarded as an emerging epidemic due to high morbidity and mortality worldwide. As the aging of the population and improving in cardiovascular event therapies, the prevalence of HF is increasing (1). Cumulative evidence demonstrated that the incidence of HF dramatically elevated in the older population (2).

Patients with HF, especially the elderly, are often accompanied by malnutrition mainly as a result of insufficient intake of micro- and macro-nutrients as well as a gradual decline in physiological and physical function. Moreover, HF and aging are also associated with a chronic low-grade pro-inflammatory status, which usually contributes to malnutrition and poor prognosis (2, 3). Thus, routine assessment of nutritional status was recommended in HF patients (4). However, evaluating the nutritional status in older patients with HF is challenging because the nutrition composition is complex, usually a combination of biological, functional, and psychological factors.

Up to now, there is no universally accepted definition of malnutrition or a gold-standard methodology for nutritional assessment in patients with HF. Several objective nutritional indexes have been testified as prognostic markers in patients with HF, such as the geriatric nutritional risk index (GNRI), prognostic nutritional index (PNI), and controlling nutritional status (CONUT) score (5–7). Among them, GNRI seems to be a relatively optimal malnutrition screening tool and showed the greatest prognostic value for outpatients with HF, possibly due to its multidimensional character containing serum markers and anthropometric factors (5).

Advanced lung cancer inflammation index (ALI), initially designed to assess the degree of systemic inflammation in metastatic non-small cell lung cancer (NSCLC) patients, has been proven to be a good predictor of adverse events in various types of cancer, Crohn’s disease, and acute decompensated heart failure as well (8–12). However, only limited research is concerned on the association of elderly HF with long-term outcomes. Due to the characteristic of increased malnutrition and inflammation level in the process of aging, we inferred that ALI might be an efficient prognostic index in elderly patients with HF. The current study aimed to explore the long-term prognostic value of ALI on admission in elderly HF inpatients, as well as compare it to GNRI, one of the well-established simply calculated nutritional indexes.

## Materials and methods

### Study population

We retrospectively enrolled HF patients above 65 years old who were admitted to the Cardiology Center of the First Affiliated Hospital of Chongqing Medical University (CQMU) between January 2015 and December 2015. HF was defined according to the recent guidelines: occurrence of HF-related symptoms or signs with evidence of cardiac dysfunction, represented by either left ventricular ejection fraction (LVEF) < 40% or elevated plasma concentration of N-terminal pro–B-type natriuretic peptide (NT-proBNP) (>125 ng/l) (13). Patients were excluded if with the following conditions: (1) lack of required data including body mass index (BMI), albumin (Alb), and neutrophil and lymphocyte count, (2) death in hospital or discharge without medical advice, (3) combined with other life-threatening diseases, (4) and lost to follow-up. The study was approved by our hospital’s ethics committee, and patients’ informed consent was waived due to the retrospective nature.

### Data collection

Baseline clinical data were collected from the patients’ medical records, including demographics, comorbidities, for example, history of hypertension, ischemic heart disease, diabetes mellitus, chronic obstructive pulmonary disease (COPD), and chronic kidney disease (CKD), smoking history, physical examination, for example, heart rate (HR) and blood pressure (BP), the New York Heart Association (NYHA) functional classification, parameters from cardiac ultrasound and electrocardiogram, and routine laboratory measurements, for example, blood count, renal and hepatic function, sodium, potassium, and NT-proBNP.

Advanced lung cancer inflammation index was derived using the following formula: ALI = BMI × Alb/NLR, where BMI = weight in kilograms/(height in meters)^2^, Alb = serum albumin in grams per deciliter, and NLR (neutrophil-to-lymphocyte ratio) = absolute neutrophil count/absolute lymphocyte count (8). While GNRI was calculated as previously reported: GNRI = 14.89 × Alb (g/dL) + 41.7 × BMI/22, where BMI/22 was set to 1 when the patient’s BMI/22 was greater than 1 (14). Patients were divided into two groups by the optimal cutoff value of ALI for predicting all-cause mortality using time-dependent receiver operating characteristic (ROC) curves.

Two endpoints were assessed during the follow-up period, including all-cause and cardiovascular mortality. Cardiovascular mortality was identified as death due to decompensated heart failure, critical arrhythmia, myocardial infarction, aortic disease, peripheral disease, or sudden cardiac death. Patients were mainly followed up by telephone contact. If no response was obtained, follow-up would be terminated at the time of the last available medical record of the patient. Patients were followed for up to 5 years.

### Statistical analysis

Continuous variables were presented as mean ± standard deviation if normally distributed or as median with interquartile range otherwise. Category variables were summarized as numbers and percentages. ALI and NT-proBNP were log-transformed as their original values were severely skewed. Comparing the baseline characteristics between the two ALI groups, the Mann–Whitney U test was adopted for continuous valuables as non-normally distributed, while the Fisher exact test or chi-square test was applied for category variables when appropriate. The Spearman rank correlation coefficient was computed to evaluate the correlation between ALI and other variables including GNRI. To evaluate the relationship between ALI and the two types of mortality, Kaplan–Meier curves were computed with event-free survival curves and compared using the log-rank test. The unadjusted univariate Cox proportional hazard regression analyses were applied to roughly show the impact of each variable on all-cause and cardiovascular mortality; then, the multivariate Cox proportional hazards analyses were performed to qualify the independent predictors for the two types of mortality. The variables contained in the multivariate analyses were those considered clinically relevant or with *p*-values less than 0.15 in univariate analyses, while BMI, Alb, and NLR were excluded due to direct correlation with ALI and GNRI. Time-dependent ROC curves were used to calculate the cutoff value of ALI, and areas under the curve (AUCs, or C-statistic) were measured for each type of mortality. We computed the net reclassification improvement (NRI) when ALI or GNRI was added to a base model containing clinical and laboratory data that was statistically significant in univariable analysis. A *p*-value less than 0.05 was considered statistically significant. All analyses were performed using the IBM SPSS Statistics version 22.0 and R version 4.2.1.

## Results

### Baseline characteristics of patients in the two groups of advanced lung cancer inflammation index

**FIGURE 1:**
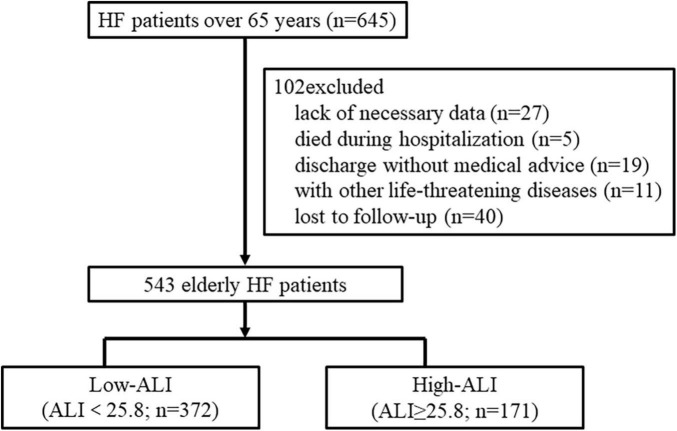
Flow chart for the study. The patients were divided into two groups based on the cutoff value of ALI for predicting all-cause mortality at 2 years. HF, heart failure; ALI, advanced lung cancer inflammation index.

During the whole year of 2015, a total of 645 patients aged over 65 years were hospitalized for HF in our center. Among them, 102 patients were excluded from the research due to the following reasons: lack of necessary data for calculating ALI or GNRI (*n* = 27), died during hospitalization or discharge without medical advice (*n* = 24), combined with other life-threatening diseases (*n* = 11), and lost to follow-up (*n* = 40). Finally, 543 elderly HF patients were enrolled in the study. The optimal cutoff value for ALI to detect all-cause mortality at 2 years by time-dependent ROC curve was 25.8 with a sensitivity of 83.5% and specificity of 37.7%. Then, the patients were categorized into two groups by ALI as follows: low ALI (ALI < 25.8; *n* = 372) and high ALI (ALI ≥ 25.8; *n* = 171) (Figure 1).

Baseline characteristics are shown in Table 1. The median age of the patients was 77 years (IQR, 71–81 years), 54.5% were women, and the median value of ALI was 18.73 (IQR, 10.43–30.09; range, 0.73–108.96). Compared to the low-ALI group, patients in the high-ALI group were younger (median age, 75 years) and had more female proportion (65.5%) and slower HR (median HR = 82 beats per minute). In addition, patients in the high-ALI group had higher values of BMI, Alb, hemoglobin, sodium, GNRI, LVEF, and better NYHA class, while they had lower values of white blood cell (WBC), NLR, aspartate aminotransferase (AST), creatinine, uric acid, NT-proBNP, and less proportion of combined CKD.

**TABLE 1:** Baseline characteristics of all patients.

	low-ALI (*n* = 372) ALI < 25.8	high-ALI (*n* = 171) ALI ≥ 25.8	*P*-value
Age, year	77 (72, 82)	75 (70, 81)	0.004
Female, *n* (%)	184 (49.5%)	112 (65.5%)	<0.001
BMI, kg/m^2^	21.8 (20, 24.6)	24.4 (21.6, 27.4)	<0.001
NYHA class II, (%)	25 (6.7%)	19 (11.1%)	0.007
III, (%)	230 (61.8%)	118 (69%)	
IV, (%)	117 (31.5%)	33 (19.3%)	
Hypertension, *n* (%)	243 (65.3%)	114 (66.7%)	0.759
Ischemic heart disease, *n* (%)	205 (55.1%)	90 (52.6%)	0.591
Diabetes mellitus, *n* (%)	115 (30.9%)	43 (25.1%)	0.169
COPD, *n* (%)	62 (16.7%)	20 (11.7%)	0.133
CKD, *n* (%)	144 (38.7%)	39 (22.8%)	<0.001
Atrial fibrillation, *n* (%)	173 (46.5%)	91 (53.2%)	0.146
Current smoking, *n* (%)	119 (32%)	41 (24%)	0.057
Heart rate,/min	88 (75, 105)	82 (72, 95)	0.008
Systolic BP, mmHg	133 (117, 154)	133 (120, 146)	0.863
Diastolic BP, mmHg	75 (66, 86)	74 (67, 86)	0.914
WBC,10^3^/μL	6.9 (5.6, 8.9)	5.5 (4.7, 6.7)	<0.001
NLR	6.1 (4.6, 8.9)	2.6 (2, 3.2)	<0.001
Hemoglobin, g/dL	12.2 (10.8, 13.4)	12.6 (11.5, 13.6)	0.024
Albumin, g/dL	3.8 (3.4, 4.1)	4 (3.7, 4.3)	<0.001
ALT, U/L	22 (14, 34)	19.5 (13, 31)	0.093
AST, U/L	27 (20, 39)	24 (18, 31)	0.013
Creatinine, mg/dL	1.1 (0.8, 1.5)	0.9 (0.7, 1.2)	<0.001
Uric acid, mg/dL	6.9 (5.4, 8.7)	6.4 (5.4, 7.5)	0.011
Sodium, mmol/L	139 (136, 141)	140 (139, 143)	<0.001
Potassium, mmol/L	4.2 (3.8, 4.7)	4.1 (3.8, 4.4)	0.067
NT-proBNP, ng/L	4,747 (2,123, 11329)	1,953 (1,077, 4,506)	<0.001
LVEF, %	54 (42, 61)	58 (46, 63)	0.005
ALI	13.6 (8.5, 19.2)	37.4 (30.7, 48.5)	<0.001
GNRI	95.3 (90.8, 99.9)	99.9 (96.8, 104.2)	<0.001

Variables are expressed as median (interquartile range) or *n* (%).

ALI, advanced lung cancer inflammation index; BMI, body mass index; NYHA, New York Heart Association; COPD, chronic obstructive pulmonary disease; CKD, chronic kidney disease; BP, blood pressure; WBC, white blood cell; NLR, neutrophil-to-lymphocyte ratio; ALT, alanine aminotransferase; AST, aspartate aminotransferase; NT-proBNP, N-terminal pro-B-type natriuretic peptide; LVEF, left ventricular ejection fraction; GNRI, geriatric nutritional risk index.

### Linear positive correlation of advanced lung cancer inflammation index with geriatric nutritional risk index

**FIGURE 2:**
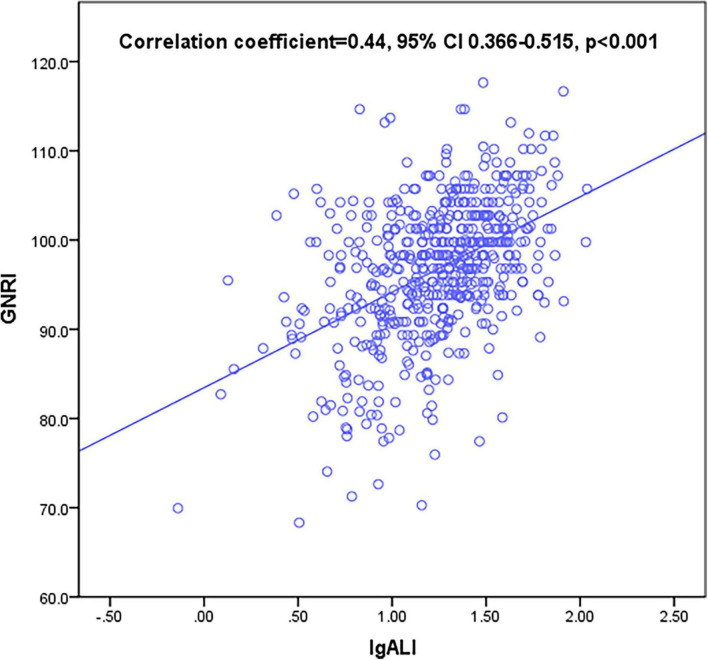
Correlation between advanced lung cancer inflammation index (ALI) and geriatric nutritional risk index (GNRI). ALI was moderately correlated with GNRI (correlation coefficient = 0.44, 95% confidential interval (CI) 0.366–0.515, *p* < 0.001).

Geriatric nutritional risk index score was regarded as a widely acceptable tool in nutrition assessment; therefore, we examined the correlation of ALI with GNRI in HF patients aged over 65 years hospitalized during the year of 2015. A correlation analysis based on Spearman’s non-parametric test has shown that the positive and linear correlation between ALI and GNRI was moderate in the total participant population [Spearman’s correlation coefficient (r): 0.44, *p* < 0.001] (Figure 2).

We also tested the correlations between ALI and other clinical, laboratory, and functional parameters to identify determinants of low-ALI values (Supplementary Table 1). It indicated that ALI also had slightly positive relations with sodium and hemoglobin values and had negative relations with a number of variables such as NT-proBNP, creatinine, the combined state of CKD, and NYHA functional class. This might suggest that low-ALI value was associated with more severe status of heart failure.

### Higher advanced lung cancer inflammation index was an independent predictor for lower all-cause and cardiovascular mortalities

**FIGURE 3:**
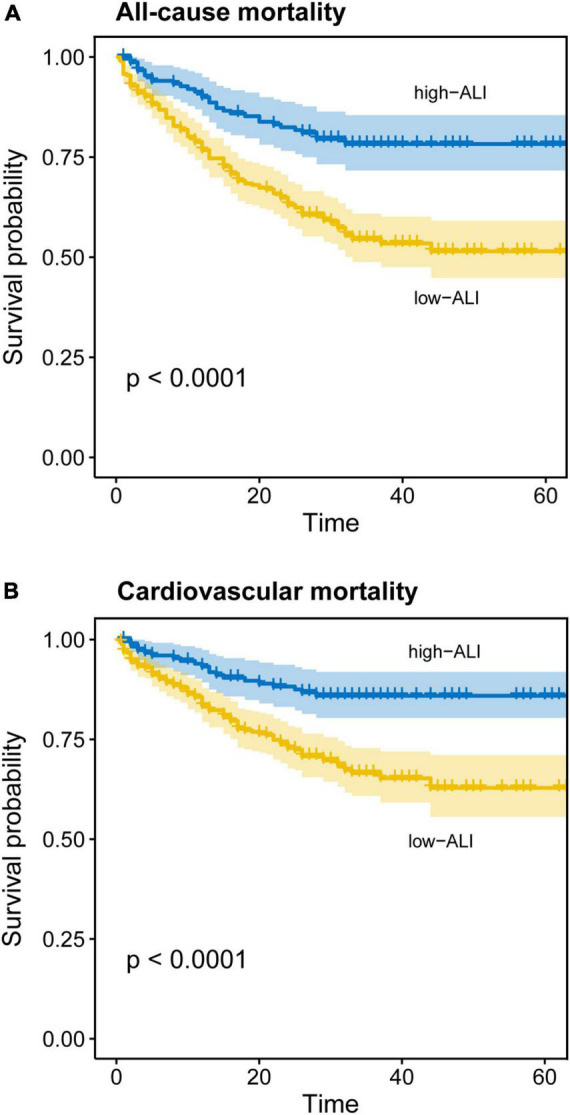
Kaplan–Meier curves for all-cause and cardiovascular mortality in elderly patients with heart failure. Cumulative event-free rate according to ALI on admission for **(A)** all-cause mortality and **(B)** cardiovascular mortality.

During the follow-up period [28(IQR 15–34) months], all-cause mortality and cardiovascular mortality occurred in 192 (35.4%) and 132 (24.3%) out of 543 patients, respectively. Cardiovascular cause accounted for 68.75% of all-cause deaths. To test the prognostic significance of ALI, unadjusted Kaplan–Meier analysis was implemented. The Kaplan–Meier analysis revealed that the cumulative incidences of both all-cause and cardiovascular mortalities were significantly higher in elderly HF patients with lower ALI (log-rank test, all-cause mortality: *p* < 0.0001; cardiovascular mortality: *p* < 0.0001) (Figures 3A,B).

In the univariate Cox proportional hazard analyses, age, gender, smoking history, COPD, CKD, NYHA, HR, systolic BP, diastolic BP, alanine aminotransferase (ALT), AST, uric acid, sodium, potassium, lg NT-proBNP, LVEF, GNRI, and lg ALI were associated with all-cause mortality (Table 2A). In terms of cardiovascular mortality, the correlated variables were almost the same as in all-cause death, except that COPD was excluded while hypertension was included (Table 2B). Furthermore, the multivariate Cox proportional hazard analyses indicated that lg ALI was an independent predictor for both all-cause (HR 0.55, 95% CI 0.349–0.867, *p* = 0.01) and cardiovascular mortality (HR 0.536, 95% CI 0.302–0.953, *p* = 0.034), while GNRI showed no independent prediction effect in the multivariate model. In addition, in the multivariate analyses, variables associated with the two types of deaths also include AST and lg NT-proBNP.

**TABLE 2:** Results of univariable analysis and final model of multivariable analysis using Cox proportional hazard analysis of all-cause death **(A)** and cardiovascular death **(B)**.

	Univariable		Multivariable	
		
	HR (95% CI)	*p*	HR (95% CI)	*p*
**(A) All-cause death**				
Age (1 year increase)	1.016 (0.994, 1.037)	0.149		
Male vs. female	1.162 (0.875, 1.542)	0.299		
Smoking	1.34 (0.995, 1.806)	0.054		
COPD	1.456 (1.017, 2.085)	0.04		
CKD	1.645 (1.235, 2.19)	0.001		
NYHA	1.467 (1.139, 1.889)	0.003		
Heart rate	0.995 (0.988, 1.001)	0.095		
Systolic BP	0.992 (0.986, 0.997)	0.004		
Diastolic BP	0.986 (0.976, 0.996)	0.007	0.983 (0.971, 0.994)	0.002
ALT	1.002 (1.001, 1.004)	0.001		
AST	1.003 (1.002, 1.004)	<0.001	1.003 (1.002, 1.005)	<0.001
Uric acid	1.092 (1.029, 1.157)	0.003		
Sodium	0.956 (0.932, 0.98)	<0.001		
Potassium	1.312 (1.101, 1.565)	0.002		
Lg NT-proBNP	2.268 (1.691, 3.043)	<0.001	1.966 (1.433, 2.695)	<0.001
LVEF	0.975 (0.963, 0.987)	<0.001		
GNRI	0.955 (0.939, 0.971)	<0.001		
Lg ALI	0.377 (0.256, 0.554)	<0.001	0.550 (0.349, 0.867)	0.01
**(B) Cardiovascular death**				
Age (1 year increase)	1.003 (0.978, 1.029)	0.804		
Male vs. female	1.396 (0.992, 1.966)	0.056		
Smoking	1.367 (0.956, 1.956)	0.087		
Hypertension	0.627 (0.444, 0.884)	0.008		
CKD	1.624 (1.15, 2.295)	0.006		
NYHA	1.663 (1.225, 2.257)	0.001		
Heart rate	0.993 (0.985, 1.001)	0.071	0.990 (0.981, 1.000)	0.045
Systolic BP	0.987 (0.98, 0.994)	<0.001		
Diastolic BP	0.987 (0.975, 0.999)	0.03		
ALT	1.003 (1.001, 1.004)	0.001		
AST	1.003 (1.001, 1.004)	<0.001	1.003 (1.001, 1.004)	<0.001
Uric acid	1.144 (1.069, 1.223)	<0.001		
Sodium	0.966 (0.935, 0.998)	0.038		
Potassium	1.32 (1.068, 1.631)	0.01		
Lg NT-proBNP	4.058 (2.782, 5.919)	<0.001	2.831 (1.782, 4.498)	<0.001
LVEF	0.953 (0.939, 0.968)	<0.001	0.976 (0.958, 0.995)	0.011
GNRI	0.957 (0.938, 0.977)	<0.001		
Lg ALI	0.344 (0.217, 0.546)	<0.001	0.536 (0.302, 0.953)	0.034

HR, hazard ratio; CI, confidence interval; COPD, chronic obstructive pulmonary disease; CKD, chronic kidney disease; NYHA, New York Heart Association; BP, blood pressure; ALT, alanine aminotransferase; AST, aspartate aminotransferase; NT-proBNP, N-terminal pro-B-type natriuretic peptide; LVEF, left ventricular ejection fraction; GNRI, geriatric nutritional risk index; ALI, advanced lung cancer inflammation index.

### Advanced lung cancer inflammation index showed non-significant improvement in risk stratification of base models

**FIGURE 4:**
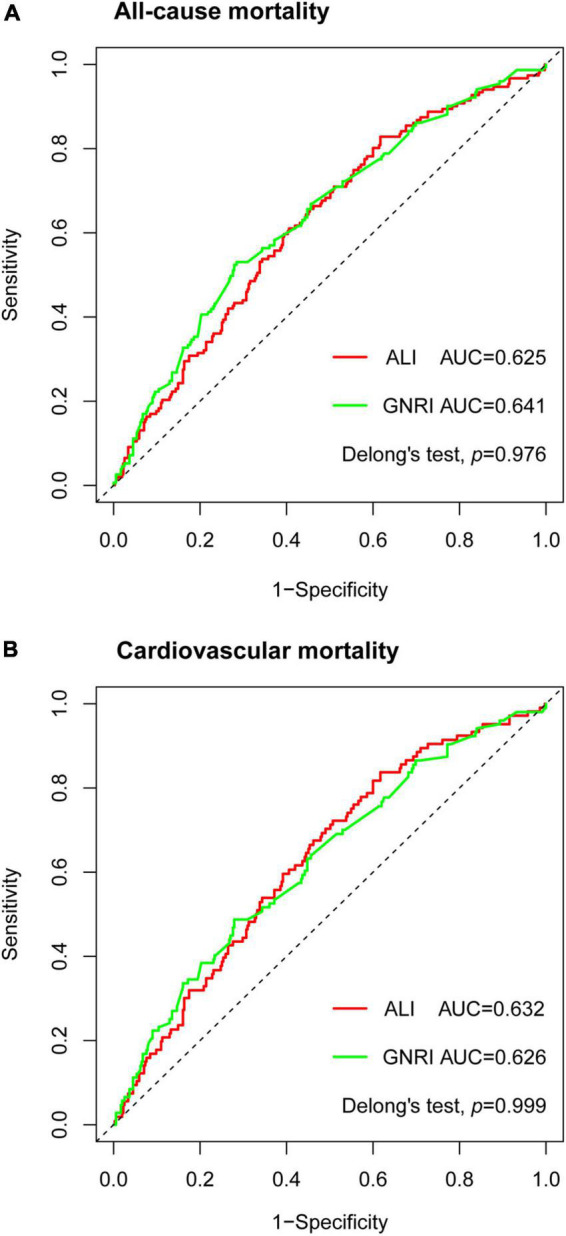
Time-dependent receiver operating characteristic (ROC) curves of advanced lung cancer inflammation index (ALI) and geriatric nutritional risk index (GNRI) with reference line for all-cause mortality **(A)** and cardiovascular mortality **(B)** at 2 years. AUC, area under the curve.

The time-dependent ROC curves demonstrated that ALI was comparable to GNRI in the prediction power at 2-year outcomes as in all-cause mortality (AUC: ALI 0.625, GNRI 0.641, *p* = 0.976) and cardiovascular mortality (AUC: ALI 0.632, GNRI 0.626, *p* = 0.999) (Figures 4A,B). We also computed the AUC of BMI, Alb, and NLR. For both two endpoints, the AUC of the three parameters was all smaller than that of ALI and GNRI (all-cause mortality, AUC: BMI 0.579, Alb 0.615, NLR 0.598; cardiovascular mortality, AUC: BMI 0.608, Alb 0.603, NLR 0.6).

To further investigate the prediction impact of ALI, we built a base model containing variables that were significant in univariable Cox proportional analyses. The base model of all-cause mortality (AUC:0.667) included COPD, CKD, NYHA, systolic BP, diastolic BP, ALT, AST, uric acid, sodium, potassium, lg NT-proBNP, and LVEF, while the base model of cardiovascular mortality (AUC:0.733) included hypertension, CKD, NYHA, systolic BP, diastolic BP, ALT, AST, uric acid, sodium, potassium, lg NT-proBNP, and LVEF. The addition of ALI resulted in non-significant improvement in risk stratification of both new models (all-cause mortality, AUC:0.674, categorical NRI 4.9%, *p* = 0.433, continuous NRI 25%, *p* = 0.022; cardiovascular mortality, AUC:0.738, categorical NRI 6.5%, *p* = 0.223, continuous NRI 27.5%, *p* = 0.029). The addition of GNRI also resulted in non-significant NRI (all-cause mortality, categorical NRI 1.9%, *p* = 0.688, continuous NRI 7%, *p* = 0.479; cardiovascular mortality, categorical NRI 2.1%, *p* = 0.533, continuous NRI 1.4%, *p* = 0.837).

## Discussion

This retrospective observational single-center study demonstrated that ALI on admission was an independent predictor of long-term all-cause and cardiovascular mortality in elderly HF patients. The correlation between ALI (contains BMI, Alb, and NLR) and GNRI (contains BMI and Alb) was only moderate, which might indicate the specific inflammation part of ALI differs from GNRI. The AUC between ALI and GNRI was comparable in predicting long-term mortalities. However, both ALI and GNRI could not significantly improve the risk stratification of base models for all-cause and cardiovascular mortalities.

Malnutrition often co-exists with HF, with a prevalence of 16 to 90% based on different nutritional screening tools (15). The incidence of malnutrition is higher in the elderly population with HF, which can be mainly attributed to insufficient nutrient intake. Malnutrition would promote the incidence and disease progression of HF (4). BMI and albumin both are routinely available markers in clinical practice and can reflect the nutritional status to some extent. However, due to the common occurrence of water-sodium retention, the BMI value in HF patients is often variational and inaccurate. Similarly, there was concern that albumin on the admission of acute HF might not be a qualified predictor of long-term prognosis due to the likely fluctuation of the value during hospitalization (16). Given the limits posed by single nutritional indicators, relatively complex indicators needing calculation were introduced in patients with HF. Several objective nutritional indices have been established in the field of HF, including the GNRI, the CONUT score, and the PNI. Among them, GNRI is calculated based on the value of albumin and BMI. A large study enrolling 3,386 outpatients diagnosed with HF has declared that malnutrition defined by either GNRI, PNI, or CONUT score, but not BMI, was common and strongly related to increased long-term mortality in this population (5). In a recent study involving 628 elderly patients with acute HF, GNRI showed slightly better performance than other nutritional indexes including CONUT and PNI in predicting in-hospital mortality (17). Evidence from a meta-analysis involving 10,589 subjects further revealed that GNRI was an independent predictor of mortality in elderly HF patients (18).

These above indexes do not directly reflect the inflammatory status in patients, while as previously mentioned, elderly HF patients are often affected by a chronic low-grade inflammation that deteriorates cardiovascular function and increases mortality (2, 19). This low-grade inflammation continues even after removing initial pro-inflammatory stimuli such as the renin–angiotensin system (RAS) and low-density lipoprotein cholesterol (LDL-C) (19).

Advanced lung cancer inflammation index was calculated as BMI × Alb/NLR and could be regarded as a nutrition-inflammation index. NLR is readily available in clinical practice and has been used as a marker of systemic inflammation. A recent multicenter study indicated that elevated NLR was an effective marker of inflammation and poor prognosis in patients with either new-onset or worsening HF, irrespective of LVEF (20).

Advanced lung cancer inflammation index was first developed as an indicator of systemic inflammation in patients with metastatic non-small cell lung cancer and was proved to be an effective predictor for mortality (8). Subsequently, the prognostic prediction effect of ALI has been testified in various types of tumors. Epidemiological studies have revealed a co-occurrence phenomenon of HF and cancer that patients with HF had a higher incidence of cancer than individuals without HF. One possible mechanism is the common pathological milieu of chronic low-grade inflammatory status that promotes both cancer and HF (3). The predictive value of ALI has also been validated in a bowel disease featured with chronic inflammatory, Crohn’s disease (CD), where ALI showed better prognostic efficiency than several other nutritional or inflammatory indexes including BMI, Alb, NLR, and PNI (11). Furthermore, Maeda et al(12). explored the prognostic value of ALI at discharge in patients with acute decompensated heart failure (ADHF) (12). During a median follow-up period of 363 days, patients in the lowest tertile of ALI had the highest incidence of primary outcome, which was a composition of all-cause mortality and readmission due to HF(12). ALI was also considered to be an independent predictor of coronary artery disease and calcification (21).

However, the long-term prognostic effect of ALI on admission in elderly HF patients remains unclear. Given that the biomarkers forming prediction indicators are not routinely tested at the time of discharge, we aimed to verify the efficiency of ALI on the time of admission for the prediction of long-term prognosis in HF patients. On the basis of the correlation between older population and chronic low-grade inflammation, we inferred that ALI could be a good prognostic index for elderly HF patients. In addition, we also followed the endpoint of cardiovascular mortality.

Our present study verified that ALI on admission was an independent predictor of long-term all-cause and cardiovascular mortality in elderly HF patients. Higher ALI was associated with significantly lower mortality. This negative correlation between ALI and mortality was consistent with the previous study concerning ALI in ADHF patients (12). In the present study, the median value of ALI was smaller than that found in the study by Maeda et al(12). (18.73 vs. 33.01). This could be explained by an older population (median age: 77-year-old vs. 75-year-old) and more female proportion (54.5 vs. 40.9%). The cutoff value of ALI to detect all-cause mortality at 2 years was 25.8. We found that elderly HF patients with ALI above 25.8 had a significantly lower mortality rate in the long-term follow-up. A moderate positive linear correlation was detected between ALI and GNRI, which might result from the sharing nutritional part while the differentiated inflammatory part between the two indicators. The time-dependent ROC analyses indicated that the prognostic efficiency of ALI was comparable to GNRI in all-cause and cardiovascular mortalities, while the NRI analyses showed that neither ALI nor GNRI could significantly improve risk stratification for long-term mortalities when added to other well-recognized clinical and laboratory predictive variables in HF.

This study had several limitations. First, the research efficiency has been limited by the single-center small-scale nature of the study. Further large-scale studies are in need to verify the prognostic value of ALI in HF patients. Second, the retrospective characteristic of the study would limit its impact. Finally, the endpoints of other major adverse cardiovascular events were not followed in the present study. Prospective studies concerning more detailed cardiovascular endpoints could be designed in future.

## Conclusion

In conclusion, ALI on admission could be a competent nutrition-inflammation marker and an independent predictor of long-term mortalities in elderly HF patients. Higher ALI was significantly associated with lower all-cause and cardiovascular mortalities in this population. However, ALI could not significantly improve risk stratification for mortalities when added to other well-recognized clinical and laboratory predictive variables in HF. These observations are of particular significance when considering the increasing elderly population in modern society.

## Data availability statement

The original contributions presented in this study are included in the article/Supplementary material, further inquiries can be directed to the corresponding author.

## Ethics statement

The studies involving human participants were reviewed and approved by Ethics Committee of The First Affiliated Hospital of Chongqing Medical University. Written informed consent for participation was not required for this study in accordance with the national legislation and the institutional requirements.

## Author contributions

XY and BH conceived the study and participated in the design. XY, BH, RW, and HT collected the data. XY, RW, and HT performed the statistical analyses. XY and BH drafted the manuscript. RW and HT helped to draft the manuscript. SL revised the manuscript critically for important intellectual content. All authors read and approved the final manuscript.
