# Supplementary material for: The prognostic value of advanced lung cancer inflammation index (ALI) in elderly patients with heart failure
Source: Front Cardiovasc Med. 2022 Nov 11;9:934551. doi: 10.3389/fcvm.2022.934551 (PMC9697177; doi:10.3389/fcvm.2022.934551)
Supplement: Supplementary file 2 [file Table_1.docx]

# Supplementary Table S1. Correlations between ALI and other clinical, laboratory and functional parameters using Spearman’s nonparametric test.

|  | r | 95%CI | *p* value |
| --- | --- | --- | --- |
| BMI | 0.345 | (0.274, 0.418) | <0.001 |
| Albumin | 0.375 | (0.291, 0.444) | <0.001 |
| NLR | -0.952 | (-0.961, -0.941) | <0.001 |
| WBC  GNRI | -0.43  0.44 | (-0.498, -0.357)  (0.366, 0.515) | <0.001  <0.001 |
| Sodium | 0.262 | (0.18, 0.339) | <0.001 |
| Hemoglobin | 0.174 | (0.089, 0.256) | <0.001 |
| NT-proBNP | -0.326 | (-0.404, -0.243) | <0.001 |
| Creatinine | -0.227 | (-0.315, -0.142) | <0.001 |
| CKD | -0.202 | (-0.285, -0.118) | <0.001 |
| NYHA | -0.17 | (-0.244, -0.097) | <0.001 |
| AST | -0.138 | (-0.226, -0.051) | 0.001 |
| Potassium | -0.13 | (-0.218, -0.043) | 0.003 |
| Age | -0.117 | (-0.202, -0.034) | 0.007 |
| Heart rate | -0.105 | (-0.191, -0.024) | 0.014 |
| ALT | -0.088 | (-0.17, -0.003) | 0.042 |
| LVEF | 0.078 | (-0.007, 0.159) | 0.074 |
| Hypertension | 0.024 | (-0.057, 0.114) | 0.573 |
| Atrial fibrillation | 0.057 | (-0.031, 0.143) | 0.187 |
| Diastolic BP | 0.029 | (-0.052, 0.117) | 0.5 |
| Male | -0.079 | (-0.161, -0.004) | 0.066 |
| Ischemic heart disease | -0.027 | (-0.108, 0.054) | 0.528 |
| Diabetes mellitus | -0.063 | (-0.15, 0.028) | 0.145 |
| COPD | -0.054 | (-0.132, 0.024) | 0.211 |
| Current smoking | -0.041 | (-0.121, 0.037) | 0.34 |
| Systolic BP | -0.014 | (-0.098, 0.075) | 0.742 |
| Uric acid | -0.077 | (-0.16, 0.008) | 0.073 |

ALI: advanced lung cancer inflammation index, r: correlation coefficient, CI: confidence interval, BMI: body mass index, NLR: neutrophil-to-lymphocyte ratio, WBC: white blood cell, COPD: chronic obstructive pulmonary disease, CKD: chronic kidney disease, NYHA: New York Heart Association, BP: blood pressure, ALT: alanine aminotransferase, AST: aspartate aminotransferase, NT-proBNP: N-terminal pro-B-type natriuretic peptide, LVEF: left ventricular ejection fraction, GNRI: geriatric nutritional risk index.
